# Early-stage serrated adenocarcinomas are divided into several molecularly distinct subtypes

**DOI:** 10.1371/journal.pone.0211477

**Published:** 2019-02-20

**Authors:** Daiki Hirano, Yuji Urabe, Shinji Tanaka, Koki Nakamura, Yuki Ninomiya, Ryo Yuge, Ryohei Hayashi, Shiro Oka, Yasuhiko Kitadai, Fumio Shimamoto, Koji Arihiro, Kazuaki Chayama

**Affiliations:** 1 Department of Gastroenterology and Metabolism, Institute of Biomedical and Health Sciences, Graduate School of Biomedical and Health Sciences, Hiroshima University, Hiroshima, Japan; 2 Department of Regeneration and Medicine Medical Center for Translation and Clinical Research, Hiroshima University Hospital, Hiroshima, Japan; 3 Department of Endoscopy, Hiroshima University Hospital, Hiroshima, Japan; 4 Department of the Faculty of Human Culture and Science, Prefectural University of Hiroshima, Hiroshima, Japan; 5 The Faculty of Humanities and Human Sciences, Hiroshima Shudo University Hiroshima, Hiroshima, Japan; 6 Department of Anatomical Pathology, Hiroshima University Hospital, Hiroshima, Japan; National Cancer Center, JAPAN

## Abstract

Serrated adenocarcinoma (SAC) is considered the end stage of the serrated neoplasia pathway. Although SAC prognosis is not widely recognized, the serrated pathway-associated subtype consistently exhibits unfavorable prognosis in genetic studies. Herein, we classified molecularly distinct subtypes of serrated adenocarcinomas and clarified their associated clinicopathological characteristics and genetic changes. We examined 38 early-stage colorectal SACs. Of these, 24 were classified into three molecularly distinct groups by colon cancer subtyping (CCS). The clinicopathological characteristics, Ki 67 labeling index (LI), and SAC epithelial serration were assessed. The DNA from carcinomas and normal tissue/adenoma was extracted by laser microdissection and sequenced by next-generation sequencing, and mutation numbers and patterns of a 15-oncogene panel were determined. The CCS groups included CCS1 (CDX2+, HTR2B-, FRMD6-, ZEB1-, and microsatellite instable-low [MSI-L]/microsatellite stable [MSS]; 14 cases), CCS2 (microsatellite instable-high [MSI-H], 5 cases), and CCS3 (CDX2-, HTR2B+, FRMD6+, ZEB1+, and MSI-L/MSS; 5 cases). Invasive cancer was significantly more frequent in CCS3 than in CCS1 (5/5 versus 3/14, respectively). Ki67 LI and epithelial serration were higher in CCS3 than in CCS1 (83.0 ± 5.8 versus 65.4 ± 4.0 and 5/5 versus 3/14, respectively; *p* = 0.031 and 0.0048). CCS2 showed the highest mutation number, whereas *KRAS* and *BRAF* mutation numbers were higher in CCS3 than in CCS1. Early-stage SACs were classified into three molecularly distinct subtypes with different clinicopathological and genetic characteristics.

## Introduction

The incidence of colorectal carcinoma (CRC) has increased rapidly during the past three decades [1]. Adenocarcinoma, the most common type of CRC, accounts for more than 90% of CRC cases [2]. In the classic genetic model for CRC tumorigenesis described by Vogelstein et al. [3], CRC evolves from adenoma to cancer as a result of genetic mutation accumulation. In this model, the adenomatous polyp is the precursor lesion of CRC [3, 4].

Recently, serrated adenocarcinomas were declared a critical element in the natural history of CRC by Jass and Smith, which typically represents the phenotype of dysplastic serrated lesion progression. The serrated carcinoma pathway is estimated to account approximately for 10–30% of all CRCs [5]. The benign serrated lesion is classified into four types: hyperplastic polyps, sessile serrated adenoma (SSA), traditional serrated adenoma (TSA), and a combination of two or more characteristics, formerly classified as mixed polyps [2, 6]. Serrated adenocarcinoma (SAC) is considered the ultimate end of the serrated pathway [7, 8]. As previously described, the morphological characteristics of SAC are defined using Mäkinen’s criteria, and include epithelial serrations, clear or eosinophilic cytoplasm, abundant cytoplasm, vesicular nuclei, distinct nucleoli, scarce (< 10%) necrosis, mucin production, and cell balls or papillary rods in the mucin. The pathological diagnosis of SAC is based on the presence of a benign serrated lesion beside the carcinoma and/or at least six of the first 7 features listed above [7].

However, the prognosis of SACs is not widely recognized. Research groups have reported that SAC is a subtype of CRC with unique histological features and a poor prognosis [7, 9, 10]. Conversely, Mäkinen et al. [6] reported no significant differences between SAC and conventional carcinoma, regarding cancer-related mortality. In comparison, our previous studies suggested that the differences in the histological and morphological types are linked to the malignant potential of SAC or benign serrated lesion [11, 12]. Recently, Lee et al. reported that serrated adenocarcinoma morphology and CpG island methylator phenotype (CIMP)-positive status are factors associated with the prognosis of advanced-stage colorectal mucinous adenocarcinoma. However, the morphology of early-stage SACs has not been fully investigated [13].

*CDX2*, a *Drosophila* caudal-related homeobox gene, is a homeobox transcription factor specifically expressed in mammalian intestinal epithelial cells; it is essential for the development, differentiation, and constant function of intestinal epithelial cells and plays a critical role in the development of the intestinal tract [14–17]. Several groups have reported that cases with CDX2- and MSS/MSI-L are associated with poor prognosis [18, 19]. Following the analysis of 469 patients with CRC, Pilati et al. reported that non-MSI-H/CDX2- patients had a very poor prognosis [19]. Notably, in the serrated pathway, the function of mutations in *BRAF* and *KRAS* is essential. The current understanding of the serrated pathway involves two main paths, i.e., *BRAF* and *KRAS* mutations. The *BRAF* and *KRAS* genes encode for kinases that belong to the mitogen-activated protein kinase (MAPK) cascades, which are involved in cell signaling that drives cell proliferation and differentiation. Mutations in the *KRAS* and *BRAF* oncogenes activates the MAPK pathway, cell proliferation, and cell survival, thereby promoting invasion and metastasis [20]. Stefanius et al. [21] reported that the frequency of *KRAS* mutation in serrated adenocarcinomas was 45.2% and suggested that the majority of *KRAS* mutants CRCs derived from benign serrated lesions. The main site for *KRAS* mutations is codon 12, and the common mutation sites are G12D, G12V, and G13D [22]. Moreover, O'Brien et al. [23] reported that the frequency of the *BRAF* mutation (V600E) in serrated adenocarcinoma is as high as 82% and that this mutation is a specific marker for the serrated pathway. Moreover, *KRAS* Q61 mutation has been reported to be associated with the primary resistance to cetuximab for CRC [24]. *BRAF* and *KRAS* are considered two of the most important genes of SACs.

Recently, genome-wide and comprehensive analyses have been widely used to analyze disease development and prognosis [25]. These studies have identified mutations, gene expression, and copy number variants that are associated with the development, growth, and invasion of CRCs [18, 26], enabling the classification of some molecularly distinct CRC subtypes. Notably, the subtype associated with the serrated pathway consistently exhibited a very unfavorable prognosis in these previous studies. Indeed, some differences in the prognosis of the serrated pathway appear to be revealed from the perspective of pathology and genetics.

Therefore, the purpose of this study was to classify the different molecular subtypes of SAC and to clarify the specific clinicopathological features and genetic changes of each subtype.

## Material and methods

### Patients

Forty patients diagnosed with early-stage colorectal SACs, selected among 1142 patients with early-stage CRC admitted at the Hiroshima University Hospital between January 2009 and January 2016, were enrolled in this study. We excluded two patients with serrated polyposis syndrome. Consequently, 38 patients with colorectal SACs were enrolled in this study. Tissue samples were collected from tissue samples stored in the pathology laboratory. This study was approved by the Independent Ethics Committee of Hiroshima University (approval number: E-1000-1; registration date: January 10, 2018). Regarding the procedure for informed consent, we provided patients with the opportunity to opt out of this research by presenting the poster on our website (https://www.hiroshima-u.ac.jp/about/public_info/other_public_info/inorinrimenu). None of the patients have opted out in this study.

### Histopathological findings

The resected specimens were fixed in 10% buffered formalin solution. The paraffin-embedded samples were then sliced into 2–3-μm-thick sections and stained with hematoxylin and eosin. The specimens were diagnosed by two pathologists (K.A. and F.S.) who were blinded to the endoscopic features of the lesion. Histological type, depth of tumor, venous invasion, and lymphatic invasion were also categorized according to the Japanese Classification of Colorectal Carcinoma [27].

When there were two or more histological types, they were classified as a dominant type. As described previously, well-differentiated tubular adenocarcinoma (tub1) was characterized by distinct and large glands, moderately differentiated tubular adenocarcinoma (tub2) was composed of medium-to-small glands with a cribriform structure, and poorly differentiated adenocarcinoma (Por) showed a limited tendency to form glands or tubules, and intracellular mucus production [27].

As previously described, the current diagnostic criteria for SAC are based on the recognition of a benign serrated lesion (hyperplastic polyp, SSA, or TSA) beside the carcinoma or characteristic carcinoma histology. The morphological characteristics of SAC were defined using Mäkinen’s criteria. The diagnosis of SAC was considered when the carcinoma met at least six of the first 7 features listed above [7] or when the carcinoma was adjacent to a benign serrated lesion [6]. Epithelial serration was characterized by epithelial tufts containing epithelium with or without basement membrane material (S1 Fig). Papillary projections with fibrovascular cores and serrate-like structures resulting from tumor cell necrosis were excluded.

### Tumor dissection and DNA extraction

First, 10-μm-thick sections obtained from paraffin-embedded samples were mounted onto polyethylene naphthalate slides (Leica Microsystems, Wetzlar, Germany), deparaffinized, and stained with hematoxylin. From these, we harvested SACs and contiguous areas of tissues using a Leica LMD 6000 laser microdissection system based on the diagnosis of the two pathologists (S2 Fig). An average of 500–1000 cells per area was collected into tubes. Finally, the DNA was extracted from the tissues collected using a GeneRead DNA formalin-fixed, paraffin-embedded (FFPE) kit (Qiagen, Hilden, Germany) according to the protocol of the manufacturer. The extracted DNA was eluted into 40 μL of buffer, quantified using Qubit HS (Thermo Fisher Scientific, Waltham, MA, USA), and stored at 4°C. The quantity of the FFPE-derived DNA samples was determined by calculating the normalized DNA integrity score (ΔΔCq) obtained by the quantitative polymerase chain reaction (qPCR) using an Agilent NGS FFPE QC Kit (Santa Clara, CA, USA).

### Microsatellite instability (MSI) analysis

The microsatellite loci (*BAT25*, *BAT26*, D2S123, D5S346, and D17S250) were amplified according to recommendations by the Bethesda guidelines [28] using the primers shown in S1 Table. The DNA extracts (2 of 50 uL) were assessed via the Multiplex-PCR approach (Toyobo, Osaka, Japan) according to the instruction of the manufacturer using an annealing Tm of 60°C. The amplification of *BAT25* and *D2S123* loci and that of *D5S346* and *D17S250* loci was combined in the duplex assays. To separate the microsatellite PCR products, the DNA 1000 LabChip Kit and Agilent 2100 Bioanalyzer were utilized according to the instructions of the manufacturer (S3 Fig). The fragment analysis was carried out using the Agilent 2100 Expert software (Agilent). To identify MSI in the SAC cases, an overlay of two electropherograms was used to compare the PCR patterns derived from tumor and nontumor tissues, as previously described [29]. The tumor MSI levels were classified into three levels based on the Bethesda guidelines [28]: MSI high (MSI-H), generally defined as MSI in more than 30% of the standard markers; MSI low (MSI-L), with changes exhibited in less than 30% of the markers; and microsatellite stability (MSS) in the absence of microsatellite alterations.

### Immunohistochemistry

Paraffin-embedded human CRC tissue was cut into 2–3 μm sections and mounted on positively charged slides. Antigen retrieval was conducted with Tris-EDTA buffer (pH 9.0) in a microwave oven at 800 W for 5 min and at 150 W for 10 min. The slides were then incubated with a primary antibody. A monoclonal mouse anti-human CDX2 antibody (M3636; Dako, Tokyo, Japan) was applied at a dilution of 1:50 for 2 h, anti-HTR2B (Sigma, HPA012867) was applied at a dilution of 1:75 for 30 min, anti-FRMD6 (Sigma, HPA001297) was applied at a dilution of 1:500 for 30 min, anti-ZEB1 (Sigma, HPA027524) was applied at a dilution of 1:500 for 30 min, anti-Ki-67 antibodies (Leica Microsystems) were applied at a dilution of 1:1000 for 30 min, anti-human CK7 antibody (M3636; Dako, Tokyo, Japan) was applied at a dilution of 1:50 for 2 h, anti-human CK20 antibody (M3636; Dako, Tokyo, Japan) was applied at a dilution of 1:250 for 2 h, anti-human MLH1 antibody (M3636; Dako, Tokyo, Japan) was applied at a dilution of 1:50 for 2 h, and anti-human p53 antibody (M3636; Dako, Tokyo, Japan) was applied at a dilution of 1:500 for 2 h; all the antibodies were applied at room temperature. The bound antibodies were detected using the EnVision system (Dako, Copenhagen, Denmark). After immunostaining, the slides were counterstained with hematoxylin. All immunohistochemical assessments were conducted without the knowledge of histological diagnoses. The Ki-67 labeling index (LI) was determined using light microscopy at the site with the highest number of Ki-67-positive cells. The cells were counted in 10 fields at 40× magnification, and the number of positive cells among 1000 tumor cells was counted and expressed as a percentage (S4 Fig).

CDX2 and ZEB1 expression level was considered positive in cases where the nuclei of cancer cells were at least, all or mostly stained. On the other hand, the cases where the nuclei of cancer cells were not stained at all or hardly stained were regarded as negative.

HTR2B and FRMD expression status was evaluated as positive in cases where the cytoplasm of the cancer cell was all or mostly stained. On the other hand, the cases where the cytoplasm of the cancer cell was not stained at all or hardly stained were considered negative [30] (S5 Fig).

The expression of both CK7 and CK20 was evaluated in the plasma membrane and cytoplasm, and less than 5% of the cells expressing these were considered to be negative. Antibody expression in 5−40% of tumor cells was considered as focused positivity; other cases were judged to be diffuse positivity (S6 Table and S6 Fig) [31]. In addition, tumors with a loss of MLH1 expression were classified as negative (S7 Fig). The p53 immunostaining results were blindly assessed with no knowledge of *TP53* aberration (S8 Fig).

### Classification of molecularly distinct subtypes

We classified 24 SAC lesions into molecularly distinct subtypes by the immunohistochemistry analysis of CDX2, HTR2B, FRMD6, ZEB1, and MSI based on the methods described by Melo et al., who reported three main molecularly distinct subtypes of CRCs using an unsupervised classification strategy involving over 1100 individuals with colon cancer [18]. The three identified subtypes (CCS1, CCS2, and CCS3) were associated with chromosomal-instability (CIN), MSI, and *BRAF* mutant/ MSS, respectively. In the study by Melo et al., the CCS3 subtype is highly related to serrated adenomas. Moreover, these three subtypes can be easily classified using the immunohistochemistry findings and by the MSI analysis [18]. Therefore, we classified 24 SAC lesions into molecularly distinct subtypes by CDX2, HTR2B, FRMD6, and ZEB1 immunohistochemistry and the MSI analysis based on the methods of Melo et al. (CCS1: CDX2 positive, HTR2B negative, FRMD6 negative, ZEB1 negative, and MSS/MSI-L, CCS2: MSI-H, and CCS3: CDX2 negative, HTR2B positive, FRMD6 positive, ZEB1 positive, and MSS/MSI-L) [18].

### Targeted enrichment and next-generation sequencing (NGS)

In all cases, 20 ng of FFPE-derived DNA was prepared for sequencing. The exons of 15 oncogenes (*NRAS*, *FOXL2*, *PIK3CA*, *PDGFRA*, *KIT*, *EGFR*, *MET*, *RET*, *KRAS*, *AKT1*, *TP53*, *ERBB2*, *BRAF*, *GNA11*, and *GNQ*) were enriched using TruSight Tumor 15 (Illumina, San Diego, CA, USA). The resulting pooled libraries were quality-controlled using Qubit HS. Sequencing was performed with paired-end reads on the MiSeq platform (Illumina).

### Variant detection

The sequenced reads were aligned to the hg19/GRCh37 reference sequence and analyzed using the MiSeq reporter (Illumina). To identify variants in SACs and contiguous areas of tissue samples, BaseSpace (Illumina) was used. The called variants were considered germline or noncancer-associated mutations if they were called in the contiguous areas of tissue samples. The remaining mutations in SAC samples were considered cancer development mutations. To reduce the false-positive rate, we set the cutoff values for SACs as follows: read depth > 100; Indel repeat length < 8; allele frequency of mutant reads > 1%; genotype quality > 30.

#### Statistical analyses

The continuous variables are presented as mean ± standard deviation. The continuous variables were compared by Student’s *t*-test, and the dichotomous variables were compared by chi-squared and Fisher’s exact tests. We assumed significance levels of 0.05 for comparisons between two groups and 0.016 (0.05/3) for comparisons among three groups. All statistical analyses were performed using R version 3.3.1.

## Results

### Purified DNA from SAC cases

We purified DNA from 38 SACs and contiguous areas of tissues but were unable to obtain sufficient quality and/or quantity of DNA from 14 cases for subsequent sequencing by NGS. Therefore, we analyzed 24 SACs with sufficiently high-quality DNA for this study.

### Clinicopathological characteristics of SACs for each molecularly distinct subtype

To determine the molecularly distinct subtypes, we classified 24 SAC cases into CCS groups based on the CDX2, HTR2B, FRMD6, and ZEB1 immunohistochemical and MSI analyses. The results of immunostaining revealed that 17 cases were positive for CDX2. Moreover, five cases were classified as MSI-H. Therefore, all 24 cases were successfully classified as molecularly distinct subtypes using CCS (CCS1: 14 cases, CCS2: 5 cases, and CCS3: 5 cases; S2 Table). Furthermore, we examined the clinicopathological characteristics, such as sex, age, tumor size, tumor depth, tumor location, macroscopic tumor type, KI67 LI, and epithelial serration, for each CCS group. The results are shown in Table 1. The incidence of T1 SACs in CCS3 (5/5) was significantly higher than that in CCS1 (3/14; *p* = 0.0048). The Ki67 LI of CCS3 tended to be higher than that of CCS1 (83.0% ± 5.8% versus 65.4% ± 4.0%, *p* = 0.031, Table 1). The frequency of tumors without epithelial serration in the SAC area in CCS1 was significantly higher than that in CCS3 and tended to be higher than that in CCS2 (*p* = 0.0048 and 0.038, respectively). Age, tumor location, tumor size, and macroscopic type did not significantly differ among the three groups.

**Table 1:** Clinicopathological characteristics of SACs for each molecularly distinct subtype.

Variable	CCS1^a^	CCS2^b^	CCS3^c^	*p*-value (a vs b, a vs c, b vs c)
Sex (male/female)	6/8	3/2	3/2	0.6285, 0.6285, 1.0
Age (years)	72.6 ± 2.6	67.6 ± 4.4	71.2 ± 4.4	0.1782, 0.3928, 0.7092
Location	5/9	4/1	4/1	0.1409, 0.1409, 1.0
(proximal/distal + rectum)				
Size (mm)	43.1 ± 6.0	28.0 ± 10.1	25.0 ± 10.1	0.1254, 0.0847, 0.3613
Depth (Tis/T1)	11/3	¼	0/5	0.0379, 0.0048, 1.0
Macroscopic type	12/2	3/2	4/1	0.2722, 1.0, 1,0
(protruded/superficial)				
Ki67 LI (%)	65.4 ± 4.0	68.0 ± 5.8	83.0 ± 5.8	0.6307, 0.9847, 0.9468
Epithelial serration	3/11	4/1	5/0	0.0379, 0.0048, 1.0
(present/absent)				

We analyzed 24 SACs that were classified into each CCS group

CCS1: CDX2+, HTR2B-, FRMD6-, ZEB1- and MSS/MSI-L, CCS2: MSI-H, CCS3: CDX2-, HTR2B+, FRMD6+, ZEB1+ and MSI-L/MSS

### Association between pathological findings and molecularly distinct subtype in T1 SACs

We investigated the histological type, SM invasive depth, budding grade, and vessel invasion of 12 T1 SAC cases in each CCS group (S3 Table). There were three, four, and five cases in CCS1, CCS2, and CCS3, respectively. The histological type of all three T1 SAC cases was differentiated adenocarcinoma. Conversely, poorly differentiated adenocarcinomas were classified as CCS3. In addition, mucinous adenocarcinomas were classified as CCS2 and CCS3. The SM invasive depth, budding grade, and vessel invasion showed no association with the CCS group.

### Examination of genetic changes in SACs and adjacent serrated lesions

We investigated the mutations in SACs and adjacent benign serrated lesion using TruSight Tumor 15 (Illumina). Initially, we searched for mutations in *KRAS* and *BRAF* of benign serrated lesions adjacent to SACs. Twenty out of 24 cases had a benign serrated lesion adjacent to the SAC. The frequency of cases with V600E or R603Q in *BRAF* in these 20 benign serrated lesions was 14% (3/21) and 4.7% (1/21), respectively. In comparison, the frequency of cases carrying mutations of G12, G13, or Q61 in *KRAS* in these cases was 38% (8/21), 24% (5/21), and 4.7% (1/21), respectively. Among the benign serrated lesions, two cases did not exhibit mutations in *KRAS* or *BRAF* (Table 2).

**Table 2:** *KRAS*/*BRAF* mutations in SACs and adjacent serrated lesions.

Case No.	Serrated lesion^ψ^	Mutation site in *KRAS* or *BRAF*		
		Adjacent serrated	SACs	
1	Yes	*KRAS* G12D	*KRAS* G12D	*KRAS* G13D
2	Yes	*KRAS* G12C	*KRAS* G12C	*KRAS* G13D
3	Yes	*BRAF* V600E	*BRAF* V600E	
4	Yes	*KRAS* G13D	*KRAS* G13D	
5	Yes	*BRAF* V600E	*BRAF* V600E	
6	Yes	*KRAS* Q61H	*KRAS* Q61H	
7	Yes	*KRAS* G13D	*KRAS* G13D	
8	Yes		*KRAS* G12D	
9	Yes	*KRAS* G12V	*KRAS* G12V	
10	Yes	*KRAS* G13C	*KRAS* G13C	
11	Yes	*KRAS* G12D	*KRAS* G12D	
12	Yes	*KRAS* G13D	*KRAS* G13D	
13	Yes	*KRAS* G13D	*KRAS* G13D	
14	Yes	*KRAS* G12V	*KRAS* G12V	
15	No			
16	Yes	*KRAS* G12D	*KRAS* G12D	
17	No		*KRAS* G12S	
18	Yes	*KRAS* G12V	*KRAS* G12V	*KRAS* D54Y
19	Yes	*BRAF* V600E	*BRAF* V600E	
20	Yes		*KRAS* G12V	
21	Yes	*KRAS* G12C	*KRAS* G12C	
22	Yes		*KRAS* Q61R	
23	No		*KRAS* G12V	
24	No		*BRAF* R603Q	

We investigated mutations in *KRAS* and *BRAF* in benign serrated lesion and SACs.

ψWhether the cases had a serrated lesion around the SAC.

**Fig 1:**
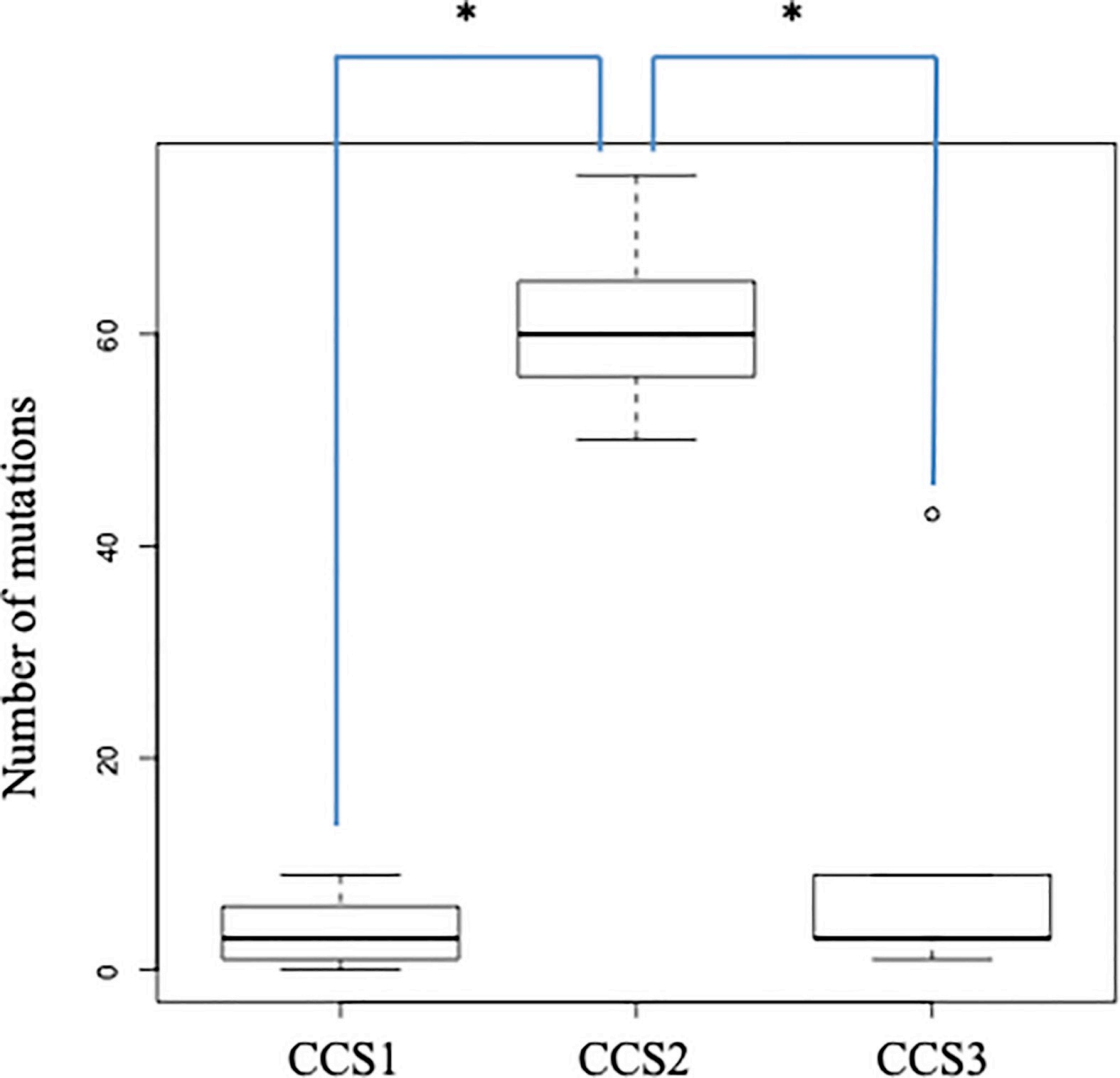
Number of accumulated gene mutations for serrated adenocarcinomas in normal tissue and adenoma-adjacent tissue in 15 cancer-related genes in each colon cancer subtyping group.

We then investigated the accumulation of mutations in SACs of normal or adenoma-adjacent tissue in 15 cancer-related genes among each CCS group (Table 2 and S4 Table). The cases that were classified as CCS2 had a higher number of mutations than those classified as CCS1 or CCS3 (median, 61.6 versus 3.1 and 11.8; *p* = 0.0001 and 0.0005, respectively; Fig 1). Moreover, examination of the frequency of gene mutation accumulation for SACs in each gene between the CCS1 and CCS3 groups demonstrated that there were no significant differences between these two groups for any of the 15 cancer-related genes (S5 Table). Because mutations in *KRAS* and *BRAF* are mutually exclusive in the serrated pathway [21], we considered the frequency of cases in which *KRAS* and/or *BRAF* mutations accumulated. There was a significant difference between CCS1 and CCS3 concerning the accumulation of *KRAS* and/or *BRAF* mutations (21.4% [3/14] versus 80.0% [4/5]; *p* = 0.038; Table 2 and S5 Table).

### Comparison between *TP53* mutations and p53-antibody immunohistochemistry

Immunostaining with p53 antibodies was performed in all 24 cases, and the positivity was evaluated and compared with *TP53* mutations. The positive cut off value was set as diffuse (> 30% staining) and null (no staining at all). Other cases were negative. (Table 3, S8 Fig). Sixteen of 24 (66.6%) lesions were positive. *TP53* mutations were identified in 14 of these 16 p53-antibody-positive lesions (87.5%). Two immunostaining-positive lesions were not identified by NGS mutational analysis. On the other hand, in 14 of 16 lesions (87.5%), *TP53* mutations were identified by NGS, and 2 of 16 lesions were p53-negative. There were no signs of staining of the background mucosa.

**Table 3:** Comparison between *TP53* mutations and p53-antibody immunohistochemistry.

Case No.	*TP53* mutation	p53 IHC
1	+	+
2	+	+
3	-	-
4	+	+
5	-	-
6	+	-
7	+	+
8	+	+
9	-	-
10	+	+
11	-	-
12	+	+
13	-	+
14	-	+
15	+	+
16	+	+
17	+	+
18	+	+
19	+	+
20	-	-
21	-	-
22	+	+
23	+	+
24	+	-

IHC; immunohistochemistry

### Comparison between CCS classification and MSI/MMR markers such as CK7, CK20, and MLH1

Immunostaining with CK20, CK7, and MLH1 antibodies was performed in all 24 cases, and the positivity was evaluated and compared with CCS classification (S6 Table, S6 and S7 Figs). Nineteen, 5, and 10 lesions of all 24 cases (79.2%, 20.8%, and 41.6%) were positively stained by CK20, CK7, and MLH1, respectively. The immunohistochemistry revealed that all carcinomas classified into the CCS1 (n = 14) and CCS3 (n = 5) groups were CK20 positive and CK7 negative. On the other hand, the IHC revealed that all carcinomas classified into the CCS2 (n = 5) group were CK20 negative and CK7 positive. This result supported that the SACs classified into the CCS2 group were MSI cases. Moreover, 2 cases in CCS1 (2/14, 14.2%), 3 cases in CCS2 (3/5, 60%), and 5 cases in CCS3 (5/5, 100%) were stained by MLH1 antibody. The frequency of MLH1 IHC positive cases in CCS2 and CCS3 was higher than that in CCS1 (*p* = 0.084, > 0.01). These results suggested that the SACs classified into the CCS2 and CCS3 groups were associated with MMR deficiency.

## Discussion

The present study demonstrated that early-stage SAC could be classified into three molecularly distinct subtypes. The histological subtype and the mutation pattern in cancerous tissue varied among subtypes. Notably, the SACs that were classified as CCS3 had several clinicopathological characteristics such as high Ki-67 LI and a high frequency of T1 carcinoma.

We showed that the molecular pathway for early-stage SACs could be classified as CIN, MSI, or one of the serrated pathways discussed by Melo and colleagues. Indeed, we presented the CDX2, HTR2B, FRMD6, and ZEB1 immunohistochemistry and MSI analyses according to the criteria defined in Melo et al. [18]. The molecular analysis revealed that, in CRC, the classical adenoma-carcinoma sequence pathway is predominantly dominated by CIN and *KRAS* mutations [4]. However, in the serrated pathway, *BRAF*/*KRAS* mutations are involved [32].

CDX2, a specific marker for colonic epithelial cells, is available for identifying colonic differentiation in metastatic adenocarcinoma [33]. In addition, it has also been shown that CDX2 suppresses CRCs [34, 35]. The majority of CRCs show strong nuclear expression of CDX2, although loss or reduction of CDX2 expression has been reported in 10−30% of colorectal cancer cases [33, 36]. Previous studies have reported that CDX2-negative colon cancer is associated with poor prognostic factors such as advanced stage, poorly differentiated cancer, vascular invasion, *BRAF* mutation, and CIMP positive status [33, 36–39]. Moreover, the lack of CDX2 expression has been reported as a poor prognostic factor for stage II and stage III CRC [30].

Microsatellite instability is caused by the loss of mismatch-repair genes, increasing the susceptibility to mutation accumulation in genes with microsatellite localization (MSI target genes) [40]. Approximately 15–20% of sporadic CRCs are reported as MSI-H. Notably, MSI-H tumor shows a genetic pathway different from that of MSS and MSI-L tumors and usually have hypermethylation of the MLH1 promoter, although there is no major chromosomal alteration [2, 41]. MSI-H is present in about 20% of SACs [21]; however, benign serrated lesions are unlikely to show high levels of MSI [42]. Generally, MSI-H CRCs have a better prognosis than common type CRCs [43].

We further examined the prognosis of early-stage SAC for each molecular subtype group. However, analysis of the prognosis of patients with early-stage carcinomas is difficult because early-stage carcinomas, such as stage Tis or T1, rarely exhibit metastasis, and a large sample set is therefore required. Consequently, we analyzed adverse prognostic variables for each subtype, which demonstrated that early-stage SAC cases classified as CCS3 (CDX2-, HTR2B+, FRMD6+, ZEB1+, and MSI-L/MSS), corresponding to the serrated pathway, were associated with adverse prognostic variables, such as strong proliferation activity, invasion into submucosa, and accumulation of *KRAS* and/or *BRAF* mutations.

Finally, we focused on the pathological findings of the CCS1 group. We found a strong association between SACs without epithelial serration in the cancerous area and cases associated with a proxy marker for CIN, which showed CDX2+, HTR2B-, FRMD6-, ZEB1-, and MSS/MSI-L. Moreover, our previous study suggested that the presence of epithelial serration in the SAC area is linked to malignant potential [11]. The pathological findings of cancerous tissue in these cases resembled common colorectal adenocarcinoma, which is closely associated with CIN. Moreover, no poor differentiated/mucinous type adenocarcinomas were included in the SACs classified into the CCS1 group (S2 Table), which suggested that it is challenging to distinguish SAC classified as CCS1 from CIN CRC using only pathological findings.

Our pilot study had some limitations. Although to the best of our knowledge, this study is the first to examine the effects of molecularly distinct subtypes on clinicopathological findings of Tis/T1 SACs, the sample size was limited. Considering the rarity of early-stage SACs, only 38 early-stage SACs were found among 1142 early-stage colorectal carcinomas examinations carried out in our Institute. To clarify the clinicopathological and genetic changes in the early stages of SAC development, we clustered SACs originating from TSA, SSA/P, and mixed-type. Therefore, it was challenging to perform subgroup analysis for genetic changes in SACs from each origin. Another limitation is the absence of a prognosis analysis. A multicenter study with a large number of cases should be conducted in the future to bypass these limitations.

In conclusion, we believe that the combination of CDX2, HTR2B, FRMD6, and ZEB1 immunohistochemistry and MSI analysis can be a prognostic factor for early-stage SACs. We believe that the information obtained by combining CDX2 immunohistochemistry and MSI analysis may contribute to the development of a treatment plan for patients with early-stage SACs.

## Supporting information

S1 Fig An example of a sessile serrated adenoma included in our study. (TIFF)

S2 Fig **Tumor was dissected by laser microdissection (bottom) from serrated adenocarcinoma (left) and benign serrated lesion (right)**.
(TIFF)

S3 Fig MSI analysis using a bioanalyzer. (a, b) Electropherograms represent the pattern of separated PCR fragments (*BAT25* and *D2S123*). (a) Due to the perfect matching pattern of both electropherograms, MSI was not determined. (b) Differences in the patterns of the two electropherograms obtained in tumor and non-tumor samples from the same patient strongly showed MSI status. (c–e) Fluorescently labeled PCR products of all five microsatellite loci (*BAT25*, *BAT26*, *D2S123*, *D5S346*, and *D17S250*) from one patient with CRC were separated by on-chip electrophoresis. Microfluidic separation of unlabeled PCR products using the Agilent 2100 bioanalyzer. Three electropherograms are shown. An overlay of the PCR pattern obtained for normal and tumorous tissues, respectively. The electropherogram overlays showed significant differences in the electrophoretic pattern of *D5S346*.
(TIFF)

S4 Fig **Photomicrographs of cases classified as CCS1 (left) with a Ki67 index of 40% and CCS3 (right) with a Ki67 index of 75%**.
(TIFF)

S5 Fig Photomicrograph of anti-CDX2, anti-HTR2B, anti-FRMD6, and anti-ZEB1 antibody immunostaining showing CCS1 (above) and negative (below) results. (TIFF)

S6 Fig Representative immunostaining of anti-human CK20 and CK7 antibodies. The top and bottom represent anti-human CK20 antibody and anti-human CK7 antibody, respectively.
(TIFF)

S7 Fig Representative immunostaining of anti-human MLH1 antibody. (TIFF)

S8 Fig Representative immunostaining of anti-human TP53 antibody. (TIFF)

S1 Table Primers used for microsatellite lesion analysis. (XLSX)

S2 Table Clinical background information for the 24 SACs that were examined in this study. We classified 24 SAC lesions into molecularly distinct subtypes using CDX2 immunohistochemistry and MSI analysis based on methods reported by De Sousa de Melo et al. [14] (CCS1:CDX+, HTR2B-, FRMD6-, ZEB1- and MSS/MSI-L, CCS2: MSI-H, CCS3: CDX-, HTR2B+, FRMD6+, ZEB1+ and MSI-L/MSS, respectively).
ΦC/C: cecum, A/C: ascending colon, T/C: transverse colon, D/C: descending colon, Ra: rectum above the peritoneal reflection, Rb: rectum below the peritoneal reflection.
ϖ MSI-L: microsatellite instability-low, MSI-H: microsatellite instability-high, MSS: microsatellite stable.
ψeEarly-stage CRCs were classied into two categories: Tis stage, carcinoma in situ; and T1 stage, tumor invasion of the submucosa.
(XLSX)

S3 Table Association between pathological findings and molecularly distinct subtypes in T1 SACs. We investigated histological type, SM invasive depth, budding grade, and vessel invasion of 12 T1 SAC cases in each CCS group.
ϖ C/C: cecum, A/C: ascending colon, T/C: transverse colon, D/C: descending colon, Ra: rectum above the peritoneal reflection, Rb: rectum below the peritoneal reflection, Rs: rectosigmoid.
ψCCS1:CDX+, HTR2B-, FRMD6-, ZEB1- and MSS/MSI-L, CCS2: MSI-H, CCS3: CDX-, HTR2B+, FRMD6+, ZEB1+ and MSI-L/MSS.
* SM invasion was measured from the lower border of the muscularis mucosae of the lesion.
ΦBudding is defined as a single cancer cell or a cluster of 5 cells along the invasion margin, and was graded per microscopic field at 200× magnification.
(XLSX)

S4 Table The accumulation of mutations in SACs of normal or adenoma-adjacent tissue in 15 cancer-related genes. We investigatedsomatic mutations of 24 SAC cases.
(XLSX)

S5 Table Comparison of mutation number between CCS1 and CCS3 for each gene. We examined the frequency of mutation accumulation from normal/adenoma tissues to cancerous tissues in each gene for SACs between the CCS1 and CCS3 groups.
ϖ; CCS1: CDX2+, HTR2B-, FRMD6-, ZEB1- and MSS/MSI-L, CCS3: CDX2-, HTR2B+, FRMD6+, ZEB1+ and MSI-L/MSS. *p < 0.05.
(XLSX)

S6 Table Comparison between CCS clasification and MSI/MMR associated IHC. CCS claficication distincted by immunohistochemistry of CDX2, HTR2B, FRMD6, and ZEB1, and MSI analysis.
(XLSX)

## Abbreviations

CCS: cancer subtyping
CIMP: CpG island methylator phenotype
CIN: chromosomal instability
CRC: colorectal carcinoma
FFPE: formalin-fixed, paraffin-embedded
LI: labeling index
MAPK: mitogen-activated protein kinase
MSI: microsatellite instability
MSS: microsatellite stability
NGS: next-generation sequencing
SAC: serrated adenocarcinoma
SM: submucosal
SSA: sessile serrated adenoma
TSA: traditional serrated adenoma
qPCR: quantitative polymerase chain reaction
