# Supplementary figures and images for: Early-stage serrated adenocarcinomas are divided into several molecularly distinct subtypes
Source: PLoS One. 2019 Feb 20;14(2):e0211477. doi: 10.1371/journal.pone.0211477 (PMC6382106; doi:10.1371/journal.pone.0211477)

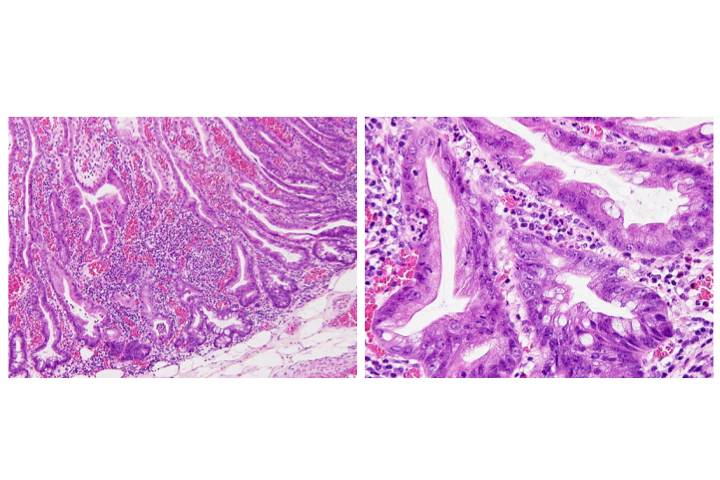

Supplement: S1 Fig — (TIFF) [file pone.0211477.s001.tiff]

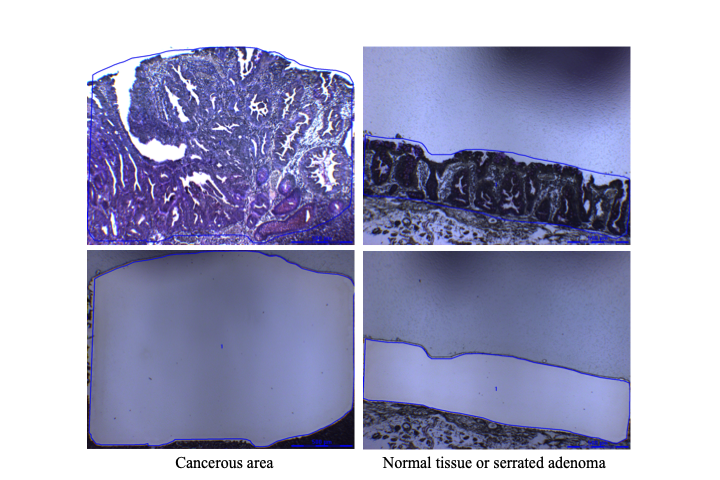

Supplement: S2 Fig — Tumor was dissected by laser microdissection (bottom) from serrated adenocarcinoma (left) and benign serrated lesion (right). (TIFF) [file pone.0211477.s002.tiff]

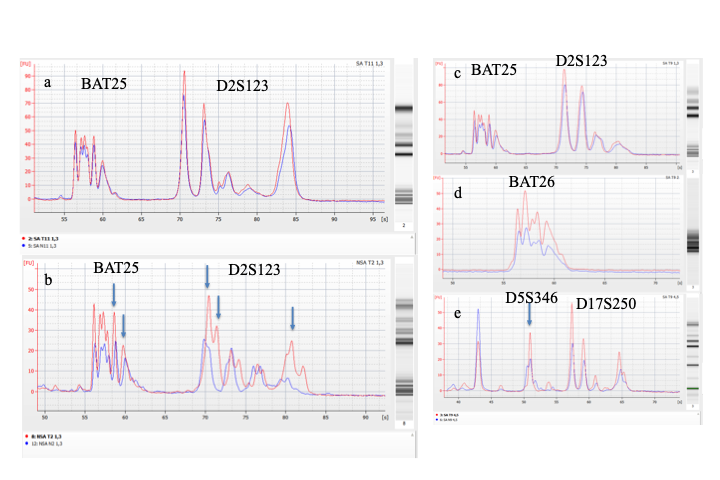

Supplement: S3 Fig — (a, b) Electropherograms represent the pattern of separated PCR fragments (BAT25 and D2S123). (a) Due to the perfect matching pattern of both electropherograms, MSI was not determined. (b) Differences in the patterns of the two electropherograms obtained in tumor and non-tumor samples from the same patient strongly showed MSI status. (c–e) Fluorescently labeled PCR products of all five microsatellite loci (BAT25, BAT26, D2S123, D5S346, and D17S250) from one patient with CRC were separated by on-chip electrophoresis. Microfluidic separation of unlabeled PCR products using the Agilent 2100 bioanalyzer. Three electropherograms are shown. An overlay of the PCR pattern obtained for normal and tumorous tissues, respectively. The electropherogram overlays showed significant differences in the electrophoretic pattern of D5S346. (TIFF) [file pone.0211477.s003.tiff]

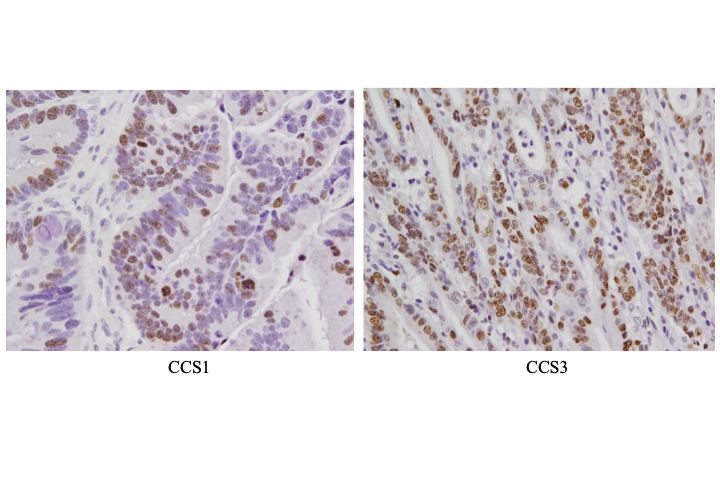

Supplement: S4 Fig — Photomicrographs of cases classified as CCS1 (left) with a Ki67 index of 40% and CCS3 (right) with a Ki67 index of 75%. (TIFF) [file pone.0211477.s004.tiff]

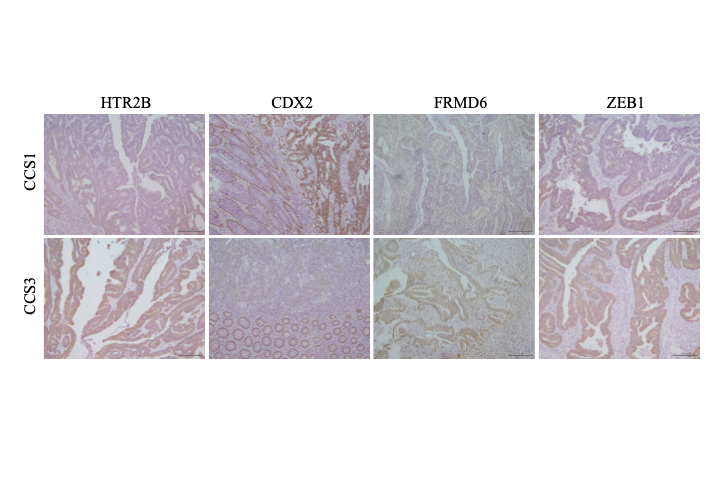

Supplement: S5 Fig — (TIFF) [file pone.0211477.s005.tiff]

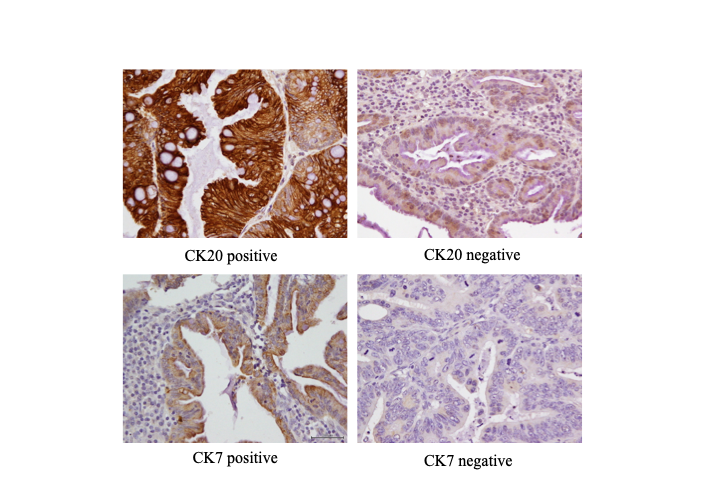

Supplement: S6 Fig — The top and bottom represent anti-human CK20 antibody and anti-human CK7 antibody, respectively. (TIFF) [file pone.0211477.s006.tiff]

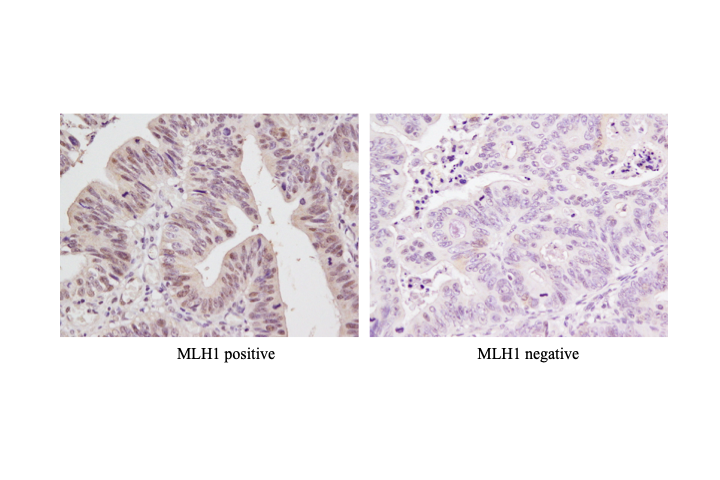

Supplement: S7 Fig — (TIFF) [file pone.0211477.s007.tiff]

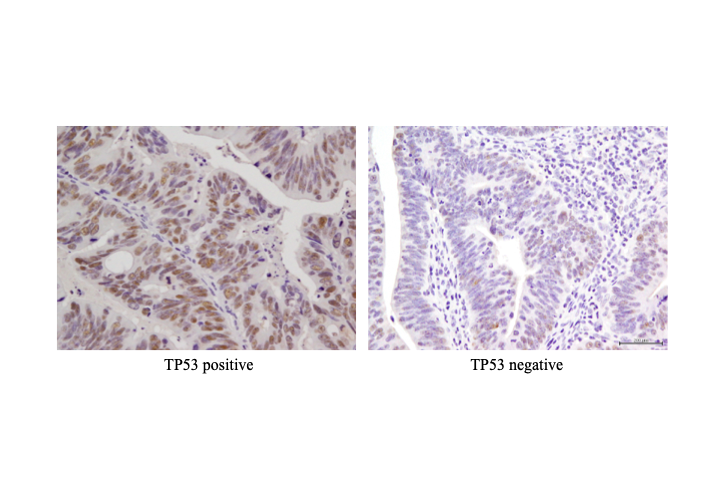

Supplement: S8 Fig — (TIFF) [file pone.0211477.s008.tiff]
